# Synthesis of copper micro-rods with layered nano-structure by thermal decomposition of the coordination complex Cu(BTA)_2_

**DOI:** 10.1186/s11671-015-0769-7

**Published:** 2015-02-05

**Authors:** Botao Qu, Xinrong Lu, Yan Wu, Xiaozeng You, Xiangxing Xu

**Affiliations:** State Key Laboratory of Coordination Chemistry, School of Chemistry and Chemical Engineering, Collaborative Innovation Center of Advanced Microstructures, Nanjing University, Nanjing, 210093 People’s Republic of China

**Keywords:** Porous metallic copper, Thermal decomposition, Lamellar layers

## Abstract

Porous metallic copper was successfully prepared by a simple thermal decomposition strategy. A coordination compound of Cu(BTA)_2_ with the morphology of micro-rod crystal was synthesized as the precursor. The precursor to copper transformation was performed and annealed at 600°C with the shape preserved. The copper micro-rods are assembled from unique thin lamellar layers, each with the thickness of approximately 200 nm and nano-pores of approximately 20 to 100 nm. This morphology is highly related to the crystal structure of the precursor. The mechanism of the morphology formation is proposed, which would be able to offer a guideline toward porous metals with controllable macro/micro/nano-structures by the precursor crystal growth and design.

**Electronic supplementary material:**

The online version of this article (doi:10.1186/s11671-015-0769-7) contains supplementary material, which is available to authorized users.

## Background

Porous metallic materials have become a burgeoning field in both applied technology and basic scientific research, especially for their significant thermal or electron conductivity, catalysis properties, importance in interface engineering, energy industry, and biomedical applications [1-4]. During the past two decades, the synthesis methods of porous metals evolved with the development of nano-science and nano-technology. Sol-gel, dealloying, and soft-template methods are typical synthetic strategies [5-8]. Among the periodic table of elements, metals of silver, nickel, copper, palladium, ruthenium, titanium, and platinum have been intensively investigated for their porous foam. For example, Walsh et al. used dextran as a sacrificial template to fabricate silver sponges, Yamauchi and co-workers prepared mesoporous nickel by an electroless deposition method in the presence of lyotropic liquid crystals, and Kuroda et al. reported 2D hexagonally ordered mesoporous metals (Ru, Pt, or Pd) by dissolving silica replica [9-11]. In addition to these methods, the combustion technology is a general method to synthesize various porous metals and oxides [12-14]. Compared with porous noble metals [15-17], porous copper was less focused, possibly for its high reactivity of oxidization in ambient atmosphere [18-26].

**Figure 1:**
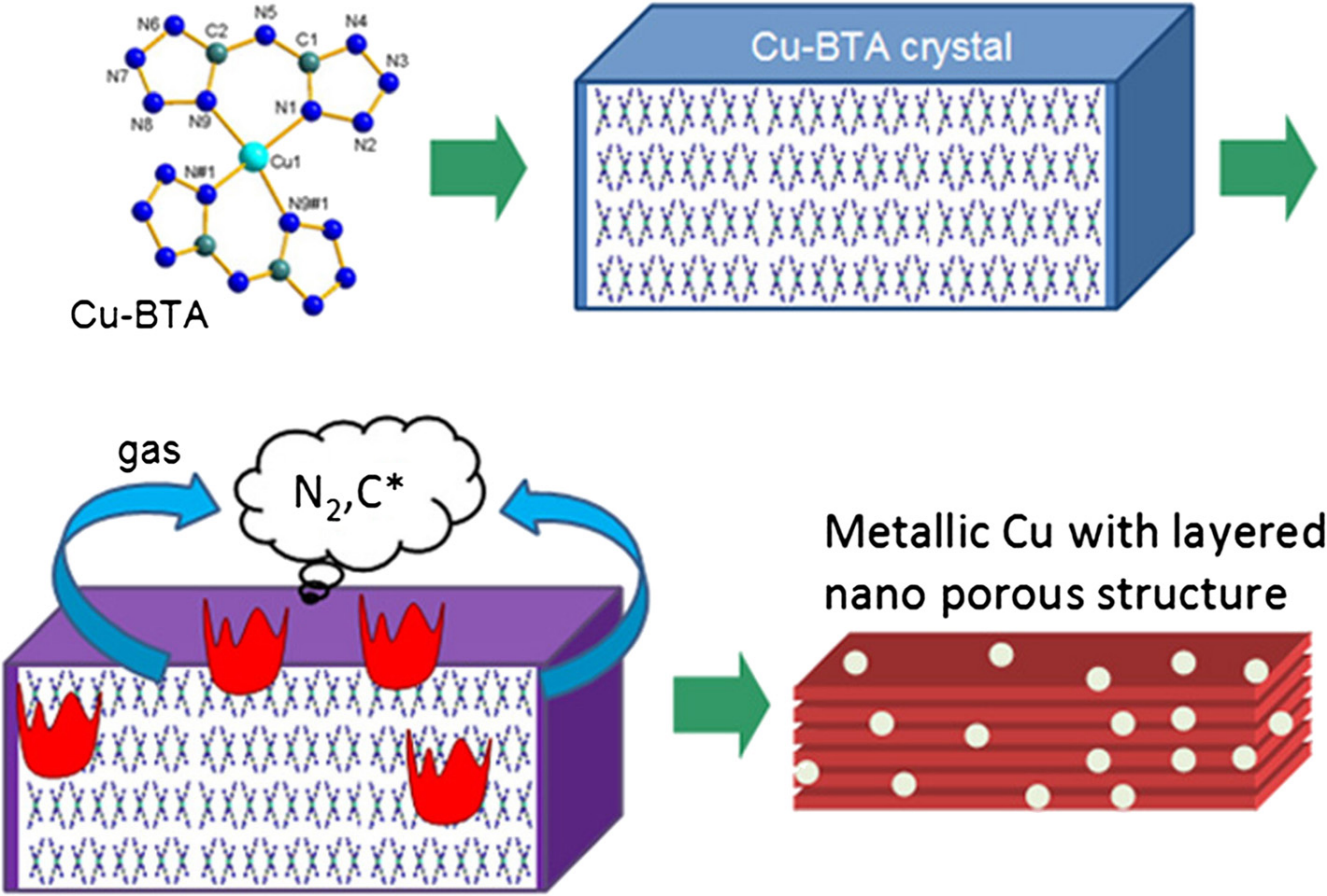
**Schematic diagram of the decomposition of a Cu(BTA)**_**2**_ **crystal to form porous copper.**

In this report, porous metallic copper micro-rods (10 to 1,000 μm) with layered nano-structure were successfully synthesized by a thermal decomposition method. Figure 1 shows the proposed synthesis diagram. The coordination compound of Cu(BTA)_2_ (BTA: bis[1(2)H-tetrazol-5-yl]amine) as the precursor was synthesized, based on the following merits. First, among various coordination compounds that contain high-nitrogen ligands, Cu(BTA)_2_ is mildly energetic during combustion process, which is favored for both the reaction and the porous structure formation [27,28]. Too high energetic precursors would destroy the porous micro/nano-structure, while less energetic precursors would lead to incomplete decomposition that induces mass impurities. Second, the absence of oxide in BTA prevents the products from oxidization. Nitrogen and carbon can be largely eliminated in the gas form from the material during the combustion process. Third, the T_d_ point group molecule configuration and crystalline morphology of the Cu(BTA)_2_ may contribute to the micro/nano-structure of the metallic face-centered cubic (fcc) copper. Therefore, the micro/nano-structure could be rationally tuned and tailored by means of a precursor molecule and corresponding crystal growth and design. Finally, in comparison with metallurgy methods, the decomposition is performed at a relatively low temperature with properties of low density and gas permeability originating from the porous feature.

## Methods

### Materials and equipment

All chemicals were purchased from commercial sources (Sigma-Aldrich, St. Louis, MO, USA) and were used without further purification. Single-crystal X-ray diffraction measurements were carried out on a Bruker SMART APEX CCD (Bruker AXS, Inc., Madison, WI, USA). Thermogravimetric analyses (TGA) were measured on a simultaneous SDT 2960 thermal analyzer with a heating rate of 20°C∙min^−1^ under N_2_ atmosphere. Powder X-ray diffraction (XRD) patterns were collected using a Bruker D8 ADVANCE X-ray diffractometer equipped with Cu-Kα radiation (*λ* = 1.5418 Å) at 40 kV and 40 mA. Scanning electron microscopy (SEM) characterizations were performed on a Hitachi S-4800 SEM (Hitachi, Ltd, Chiyoda-ku, Japan), equipped with energy-dispersive X-ray spectroscopy (EDS). The transmission electron microscopy (TEM) images were obtained from JEOL JEM-2100 (JEOL Ltd., Akishima-shi, Japan) operating at 200 kV. The electronic semiconducting property f the samples was recorded by the semiconductor device analyzer B1500A from Agilent Technologies (Santa Clara, CA, USA). Elemental analysis is measured by Elementar vario MICRO (Hanau, Germany). The Fourier transform infrared (FTIR) spectrum was measured by a VECTOR 22 spectrometer with KBr pellets (Bruker AXS, Inc., Madison, WI, USA).

### Synthesis

A mixture of CuCl_2_ · 2H_2_O (AR, 0.1 mmol), BTA (99%, 0.1 mmol), and NH_3_ · H_2_O (AR, 25 to 28 wt%, 1.0 ml) in CH_3_CN (AR, 5 ml) was sealed in a Teflon-lined stainless steel autoclave and heated to 100°C under autogenous pressure for 48 h. When cooled to room temperature, blue rodlike Cu(BTA)_2_ crystals were isolated. They were rinsed with CH_3_CN and dried in vacuum at 60°C overnight. The Cu(BTA)_2_ was decomposed and annealed in an Ar flow atmosphere at 600°C to yield the porous metallic copper.

## Results and discussion

Single-crystal X-ray diffraction reveals that the crystal structure of the precursor belongs to the monoclinic space group *C*2/*c* (Figure 1). The mononuclear complex contains one four-coordinated Cu(II) cation and two BTA anions with the formula Cu(BTA)_2_. Each of the BTA ligands provides two donor N atoms to coordinate to the Cu(II) ions, exhibiting a distorted tetrahedron coordination geometry (Additional file 1: Figure S1). The Cu-N bond lengths for each ligand are 1.954 and 1.969 Å, and the dihedral angle of two BTA planes is 36.35°. The CCDC no. 1035211 contains the supplementary crystallographic data for the Cu(BTA)_2_ complex (Additional file 2).

**Figure 2:**
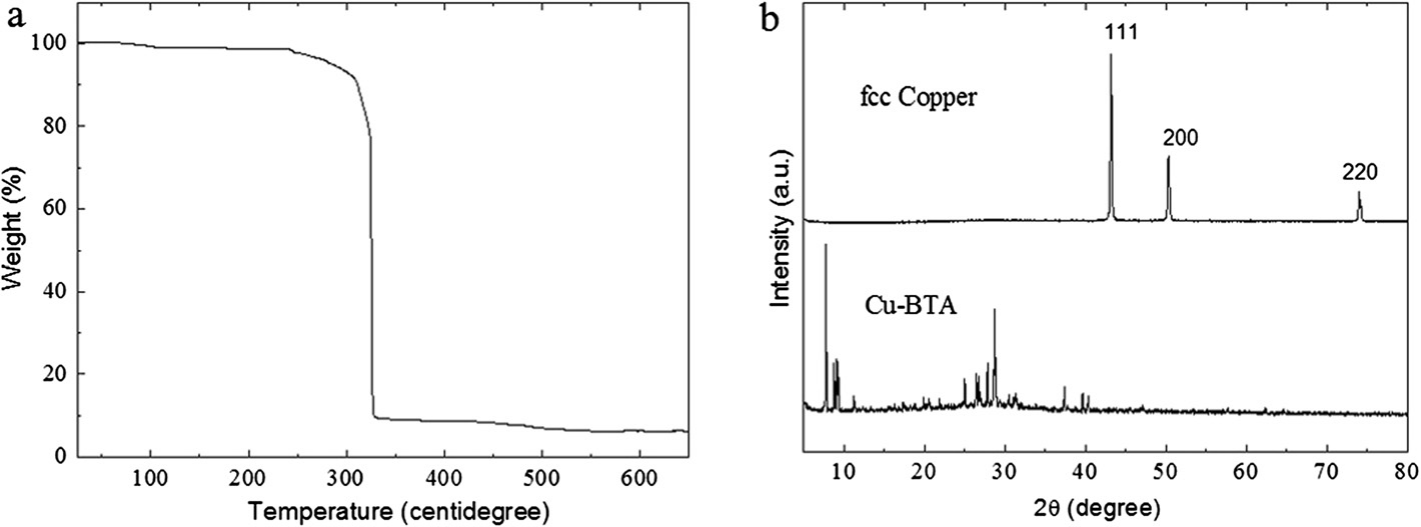
**TGA curve and XRD patterns. (a)** TGA curve of the Cu(BTA)2 precursor. **(b)** XRD patterns of the Cu(BTA)_2_ crystals and the copper product.

The TGA curve of the Cu(BTA)_2_ complex shows that there exist clear stages for the thermal decomposition. The material remains stable until the temperature rises to 240°C that the compound becomes decomposed. It undergoes a steep weight loss at 325°C and ends at approximately 340°C (Figure 2a). A minor weight loss was observed within 400°C to 550°C, which could be due to the further decomposition of carbon by-products [28]. It means that the precursor can be ignited at a temperature as low as 325°C to 340°C. The annealing temperature of 600°C was used to achieve higher product purity. During the self-sustained combustion, the Cu^2+^ ions are reduced to metal Cu^0^ atoms. These atoms agglomerate into small grains, which assemble into large structures along with the generation of N_2_- and carbon-containing gases.

The XRD measurement reveals that after the decomposition at 600°C, Cu(BTA)_2_ completely transformed into metallic copper. The diffraction peaks (Figure 2b) can be indexed to (111), (200), and (220) crystal planes of fcc copper (pdf #88-1326). The original morphology of the Cu(BTA)_2_ crystals was largely inherited by the metallic copper, showing a short rodlike shape, while at the same time, remarkable size shrinkage (up to approximately 90% volume shrinkage) is observed for the metallic copper compared with the Cu(BTA)_2_ crystal precursor.

**Figure 3:**
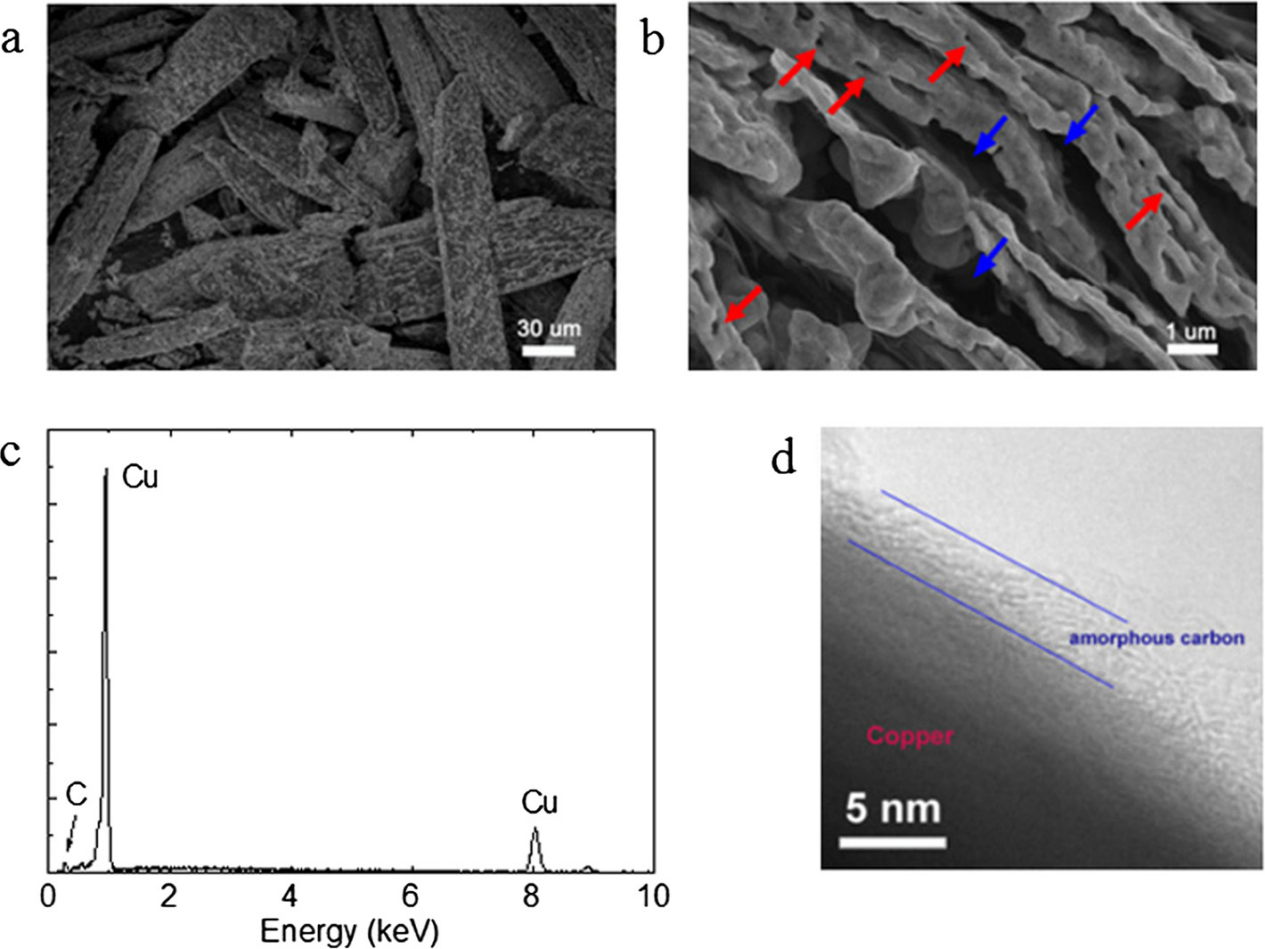
**SEM images, EDS spectrum, and TEM image. (a, b)** SEM images of the porous copper layered structure. Red arrows: small pores; blue arrows: gaps between layers. **(c)** EDS spectrum. **(d)** TEM image of the copper sample.

The porous feature is confirmed by the SEM measurement. Typical SEM images are shown in Figure 3a,b. A unique nano-structure was observed for the sample. All these copper micro-rods are assembled by loosely piled nano-sheets with the thickness approximately 200 nm. The gaps between the nano-sheet layers are approximately 0.5 to 2 μm. Nano-pores of the size approximately 20 to 100 nm are also observed within the nano-sheets. The porous structures - both the micro-gaps and nano-pores - are believed to be induced by the N_2_- and carbon-containing gases generated during the decomposition. Interestingly, the piled copper nano-sheets are well arranged parallel to the axial direction. The EDS spectrum showed that the copper product is mainly composed of copper and some minor carbon (Figure 3c). The minor carbon should be from the residual by-products in the decomposition procedure. Elementary analysis shows that the C content is 0.59 wt%. The TEM measurement confirms that a thin layer (approximately 4 nm) of amorphous carbon exists in the outer layer of the copper (Figure 3d). It is still unclear if or to what extent the carbon exists in the copper crystal in the form of an interstitial solid solution.

**Figure 4:**
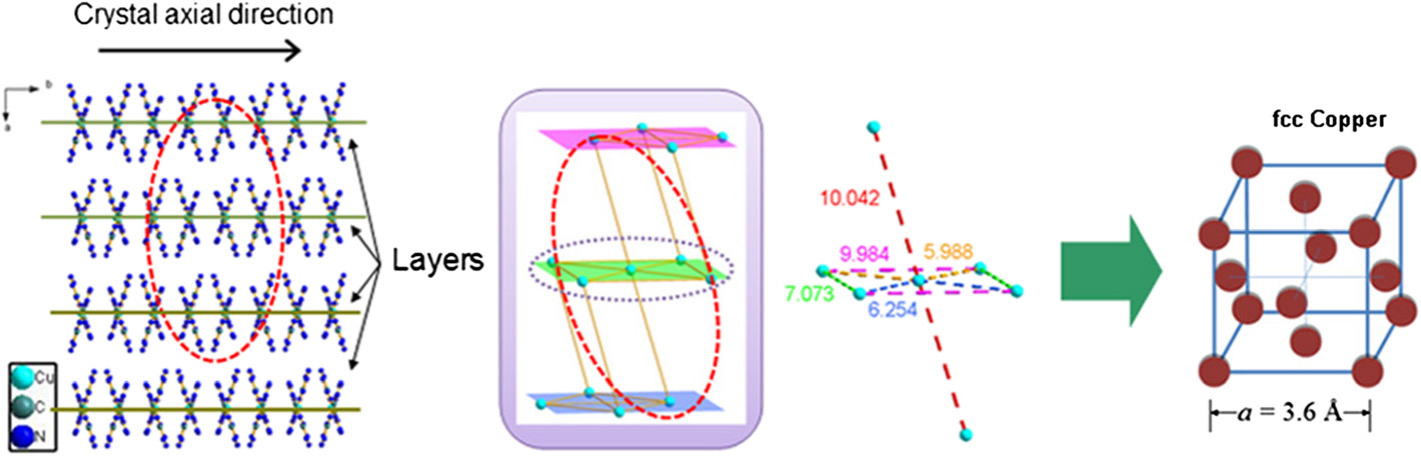
**Schematic illustration of the Cu(BTA)**_**2**_ **crystal structure and copper transformation.**

To understand how this orientation is formed, we take a closer look at the Cu(BTA)_2_ crystal structure (Figure 4). The Cu(BTA)_2_ micro-rods grow in the [010] direction. In the (100) crystal plane, the adjacent Cu-Cu distances are, respectively, 5.9, 6.2, and 7.0 Å, while in the [100] direction, the Cu-Cu distance is as large as 10.2 Å. Therefore, the Cu(BTA)_2_ presents a layered crystal structure. This layered structure matches well with the layered structure of the corresponding copper. From this clue, the mechanism of the copper micro/nano-structure formation is suggested. The decomposition of each Cu(BTA)_2_ molecule generates a single Cu^0^ atom at the position *in situ*. These isolated Cu^0^ atoms are highly active to attract each other forming a Cu-Cu metallic bond. The Cu-Cu metallic bond has the length of 3.6 Å, smaller than the Cu-Cu distance in the precursor. So the Cu^0^ atoms would preferably approach from the short distance in a statistical thermodynamic way, that is, in the (100) crystal plane of the precursor. Thus, it intends to form a Cu layer structure with the thickness in one-atom scale. However, as the Cu-Cu bond formed, it obeys the lowest energy rule to transform into the fcc phase instantly. These initially formed Cu nano-clusters undergo alloying to form bigger crystals. The degassing process during the decomposition may contribute to the layered nano-structure: the decomposed N_2_- and carbon-containing gases would escape preferably through the gaps between the layers, which may generate oriented pressure to shape the metallic copper.

**Figure 5:**
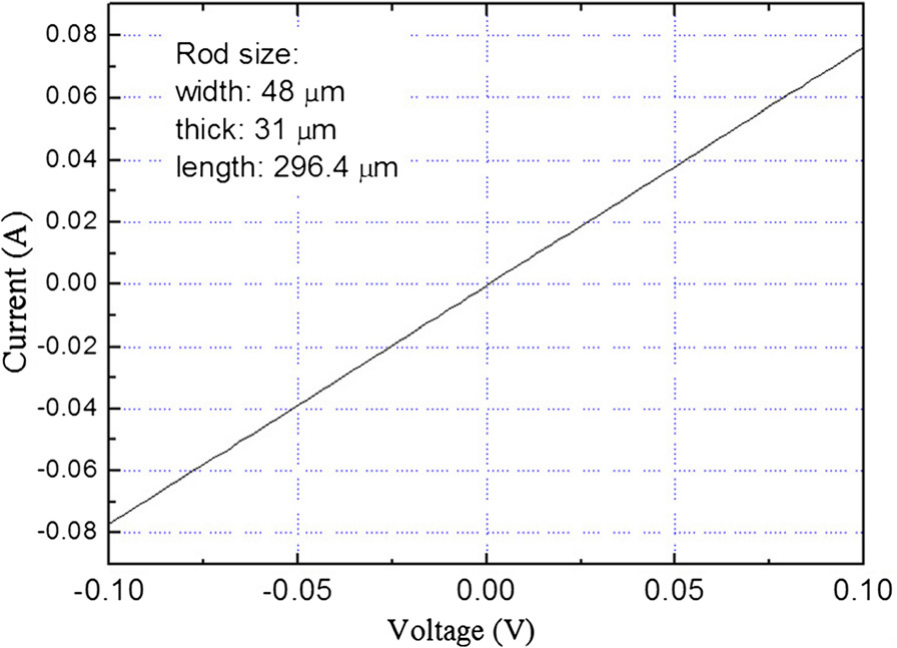
***I*** **-** ***V*** **measurement of a copper micro-rod along the longitudinal direction.**

The electrical conductivity of one Cu micro-rod was measured at room temperature (Figure 5). The electric conductivity is approximately 10^5^ S∙m^−1^ in the longitudinal direction. It is higher than amorphous carbon (approximately 2 × 10^4^ to 3 × 10^4^ S∙m^−1^) and lower than pure copper (5.96 × 10^7^ S∙m^−1^) [29], which is consistent with the theory of the electrical conductivity of composites [30]. In comparison, the copper micro-rod sample was further pressed into a rectangular block with the size of 0.888 × 4 × 12 mm under 8 Mpa for 5 min. The electrical conductivity was found to be 7.4 × 10^6^ S·m^−1^, which is one order of magnitude higher than that of the Cu micro-rod. It indicates that the electrical property depends not only on the component fractions, size, and shape but also on the porous structure: when intensively pressed, the pores are eliminated, leading to the enhancement of the electrical conductivity [30]. The micro/nano-porous structure and good conductivity suggest that the material may have application potentials in batteries/cells [31-33], sensing devices [34], and catalysis [35].

## Conclusions

In summary, we report a thermal decomposition method to prepare copper micro-rods with layered porous structure for the first time, by using a well-designed coordination compound of Cu(BTA)_2_ crystal as the precursor. The shape of the crystals was preserved for the copper product. This allows us to obtain copper with various morphologies by the growth of the precursor crystals of different sizes and shapes, without changing the molecule itself. Moreover, the layered nano-structure is highly related to the crystal parameters of the precursor. It could be expected that the same precursor crystallizes in different crystalline spaces; accordingly, the micro/nano-structure would be tuned.

## Additional files

Additional file 1: Figure S1. The dihedral angle of two BTA planes in a Cu(BTA)_2_ molecule. **Figure S2.** FTIR spectrum of the Cu(BTA)_2_ precursor. Additional file 2:
**The crystallographic information file of the Cu(BTA)**_**2**_ **complex.**
