# Supplementary material for: Synthesis of copper micro-rods with layered nano-structure by thermal decomposition of the coordination complex Cu(BTA)2
Source: Nanoscale Res Lett. 2015 Feb 5;10:42. doi: 10.1186/s11671-015-0769-7 (PMC4384981; doi:10.1186/s11671-015-0769-7)
Supplement: Additional file 1: Figure S1. — The dihedral angle of two BTA planes in a Cu(BTA)2 molecule. Figure S2. FTIR spectrum of the Cu(BTA)2 precursor. [file 11671_2015_769_MOESM1_ESM.doc]

Supplementary Information

**Synthesis of copper micro-rods with** **layered nano structure by thermal decomposition of the coordination complex Cu(BTA)_2_**

Botao Qu, Xinrong Lu, Xiaozeng You**^＊^**, and Xiangxing Xu**^＊^**

State Key Laboratory of Coordination Chemistry, School of Chemistry and Chemical Engineering, Collaborative Innovation Center of Advanced Microstructures, Nanjing University, Nanjing 210093, P. R. China.

* Correspond to: youxz@nju.edu.cn; xuxx@nju.edu.cn


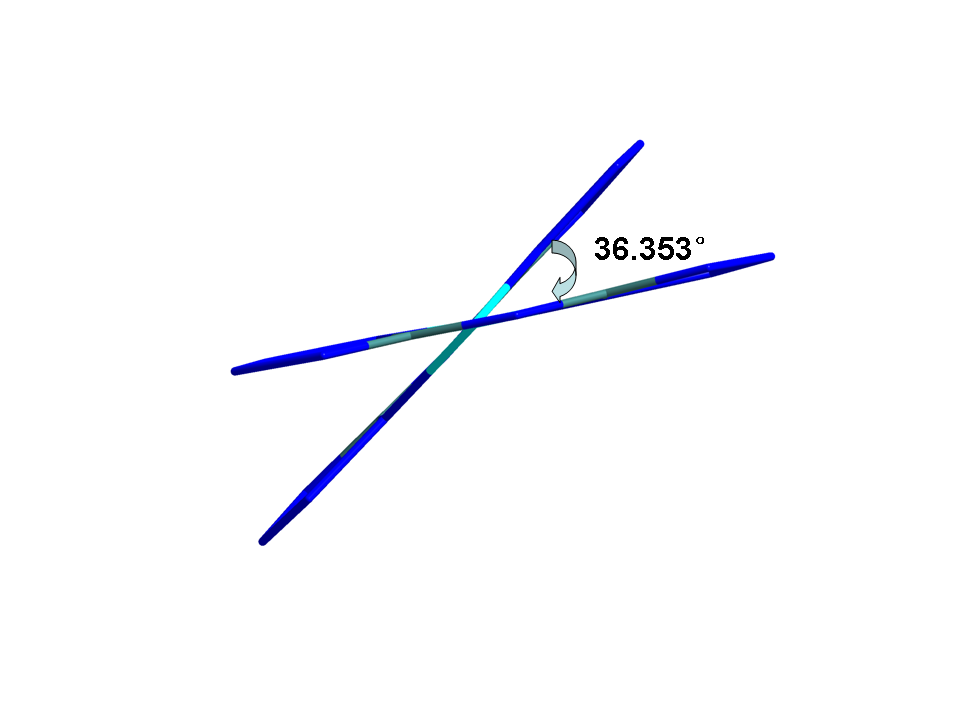


**Figure S1** The dihedral angle of two BTA planes in a Cu(BTA)_2_ molecule.


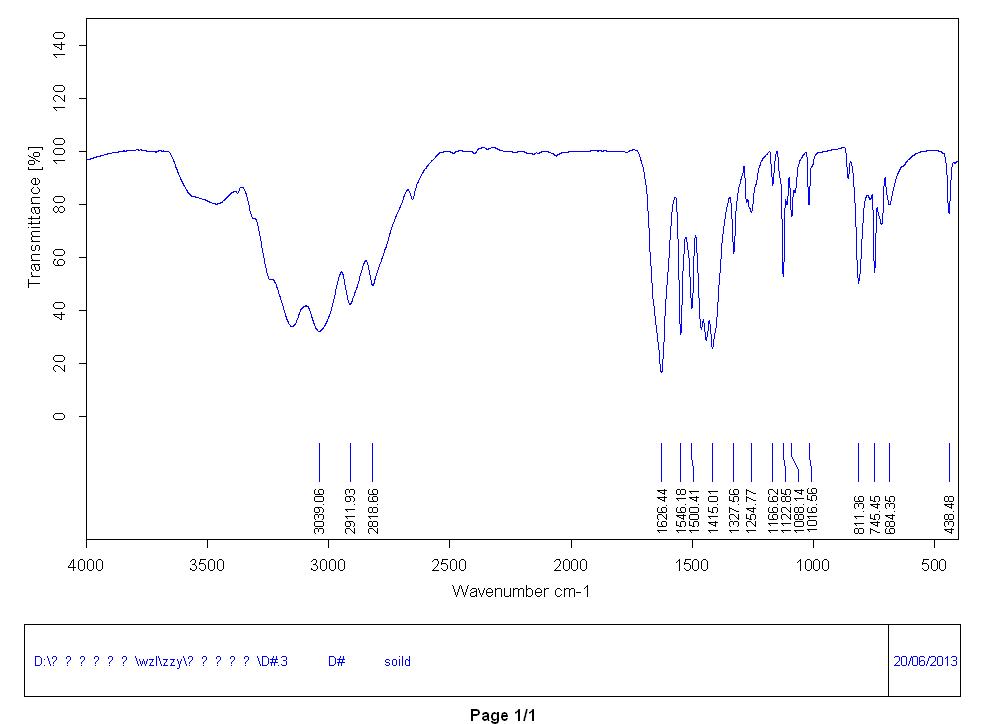


**Figure S2** FTIR spectrum of the Cu(BTA)_2_ precursor.
